# A Brief Review on the Regulatory Roles of MicroRNAs in Cystic Diseases and Their Use as Potential Biomarkers

**DOI:** 10.3390/genes13020191

**Published:** 2022-01-22

**Authors:** Luis M. Ruiz-Manriquez, Schoenstatt Janin Ledesma Pacheco, Daniel Medina-Gomez, Andrea G. Uriostegui-Pena, Carolina Estrada-Meza, Anindya Bandyopadhyay, Surajit Pathak, Antara Banerjee, Samik Chakraborty, Aashish Srivastava, Sujay Paul

**Affiliations:** 1 Tecnologico de Monterrey, School of Engineering and Sciences, Campus Queretaro, Av. Epigmenio Gonzalez, No. 500 Fracc. San Pablo, Queretaro 76130, Mexico; a01701195@itesm.mx (L.M.R.-M.); A01700204@tec.mx (S.J.L.P.); A01701230@tec.mx (D.M.-G.); A01700224@tec.mx (A.G.U.-P.); A01272850@itesm.mx (C.E.-M.); 2 C4 Rice Center, International Rice Research Institute, Manila 4031, Philippines; Anindya.B@ril.com; 3 Synthetic Biology, Biofuel and Genome Editing R&D, Reliance Industries Ltd., Navi Mumbai 400701, India; 4 Department of Medical Biotechnology, Chettinad Academy of Research and Education (CARE), Chettinad Hospital and Research Institute (CHRI), Faculty of Allied Health Sciences, Chennai 603103, India; surajit.pathak@gmail.com (S.P.); antara.banerjee27@gmail.com (A.B.); 5 Division of Nephrology, Boston Children’s Hospital, Harvard Medical School, Boston, MA 02115, USA; samik.chakraborty@childrens.harvard.edu; 6 Department of Clinical Science, University of Bergen, 5021 Bergen, Norway; aashish.srivastava1302@gmail.com

**Keywords:** microRNAs, cystic diseases, polycystic ovarian syndrome, polycystic kidney disease, pancreatic cyst tumors, biomarkers

## Abstract

miRNAs are small endogenous conserved non-coding RNA molecules that regulate post-transcriptional gene expression through mRNA degradation or translational inhibition, modulating nearly 60% of human genes. Cystic diseases are characterized by the presence of abnormal fluid-filled sacs in the body, and though most cysts are benign, they can grow inside tumors and turn malignant. Recent evidence has revealed that the aberrant expression of a number of miRNAs present in extracellular fluids, including plasma or serum, urine, saliva, follicular fluid, and semen, contribute to different cystic pathologies. This review aims to describe the role of different miRNAs in three worldwide relevant cystic diseases: polycystic ovarian syndrome (PCOS), polycystic kidney disease (PKD), and pancreatic cyst tumors (PCTs), as well as their potential use as novel biomarkers.

## 1. Introduction

Cysts are sacs or capsules that generally contain a liquid or semisolid substance. They vary in size and can be originated anywhere in the body, such as skin, ovaries, breasts, and kidneys, among other organs [1,2]. Although the majority of cysts are benign, they can also form inside tumors and become cancerous. The most common causes of cyst formation are clogged sebaceous glands, duct blockages, injuries that cause vessels to burst, tumors, genetic disorders, chronic inflammatory diseases, cell defects, parasitic organisms, and infections [1,2,3]. The symptoms differ greatly depending on the type and location of the cyst. In most cases, the patient notices an unusual lump beneath the skin, while in other cases, patients might experience headaches if the cyst is in the brain or localized pain. Internal cysts can only be detected with imaging scans [3]. It has been well elucidated that the dysregulation of crucial molecular signaling pathways induces cyst formation [4], while abnormal epithelial proliferation and apoptosis promote cyst expansion [5]. The injury-induced acceleration of cystogenesis (formation of a cyst) is most likely the result of a combination of processes such as cell death, proliferation, epithelial dedifferentiation, altered planar cell polarity, and inflammation [6].

Polycystic ovarian syndrome (PCOS) is a condition characterized by hyperandrogenism, ovulatory dysfunction, and polycystic ovarian morphology [7], and is considered to be a complex multigenic disorder with epigenetic and environmental influences [8]. It is the most common endocrine-metabolic disorder in women of reproductive age [9]. In comparison, Polycystic kidney disease (PKD) is distinguished by enlarged kidneys and weakened renal function [10], as well as fibrosis and destruction of normal renal parenchyma as the disease progresses [11]. PKD is a common genetic disorder (affecting ~4% of people worldwide) [12] that is triggered by mutations in numerous genes and is a common cause of end-stage renal disease [10]. On the other hand, Pancreatic cyst tumors (PCTs) are extremely common, accounting for between 2.4 to 13.5% of abdominal imaging findings [13], and could be benign or malignant [14]. However, the only current treatment option is surgical excision, associated with significant morbidity and mortality [13]. Given the large number of people affected by these diseases, it is critical to conduct research on them to understand the underlying molecular mechanism for developing novel biomarkers and therapeutic strategies.

**Figure 1:**
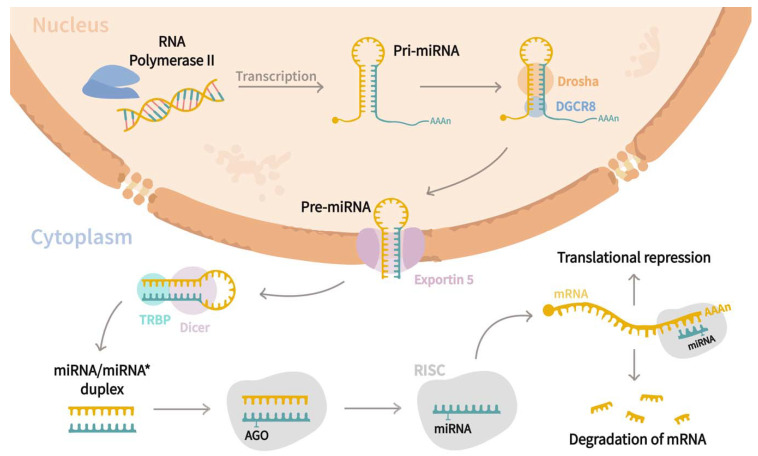
miRNAs biogenesis pathway. Canonical biogenesis pathway begins in the nucleus, where miRNA genes are transcribed as an extended hairpin shape called primary miRNA (pri-miRNA) by the RNA polymerase II. Later, a microprocessor complex composed by Drosha, DiGeorge syndrome critical region eight genes (DGCR8), and associated proteins cleaves both strands of the loop, yielding a shorter stem-loop structure of 60 to 70 nucleotides called precursor miRNA (pre-miRNA). Following that, the pre-miRNA is exported to the cytoplasm by Exportin-5, where it is processed by the RNase III endonuclease Dicer and the RNA-binding protein TRBP, which trim the loop to form a miRNA/miRNA* duplex. The duplex is then inserted into the RNA-Induced Silencing complex (RISC) under the guidance of an argonaute (AGO) protein, where helicase separates the strands, and one of them constitutes the mature miRNA. Finally, the RISC–mRNA complex recognizes specific mRNA by sequence complementarity, resulting in mRNA degradation or translational inhibition.

MiRNAs are a family of small (21–25 nucleotides in length) non-coding regulatory RNAs that control the expression of genes at the post-transcriptional level [15,16,17,18]. Since their discovery in *Caenorhabditis elegans* by Lee et al. [19], they have been found to be widely distributed in most of the eukaryotic organisms [20], and to date, ~2300 mature miRNAs have been associated with humans regulating almost 60% of genes [21,22,23,24,25]. Biogenesis of miRNA (Figure 1) starts in the nucleus, where RNA polymerase II transcribes miRNAs genes as an extended hairpin structure called primary miRNA (pri-mRNA). After that, the microprocessor complex formed by Drosha, DiGeorge syndrome critical region eight gene (DGCR8), and associated proteins, cleave both strands of the loop resulting in a shorter stem–loop structure of 60 to 70 nucleotides long, named precursor miRNA (pre-miRNA). Subsequently, pre-miRNA is exported to the cytoplasm through the action of Exportin-5, where its maturation is carried out by the RNase III endonuclease Dicer and RNA-binding protein TRBP, which trim the loop resulting in a miRNA/miRNA* duplex. The duplex is further incorporated into the RNA-Induced Silencing complex (RISC) guided by an argonaute (AGO) protein, where strands are separated by helicase, and one of them becomes the mature miRNA. Afterward, the RISC-mRNA complex recognizes specific mRNA sequences by base complementarity, resulting in mRNA degradation or translational inhibition [20,21,23,26]. Interestingly, it has been stated that a single transcript might be targeted by multiple miRNAs, while a sole miRNA can regulate up to 200 different mRNAs [15,24,27]. miRNAs are involved in a wide range of biological functions, including cell proliferation, differentiation, and apoptosis [28,29], and those are crucial to the pathogenesis of cystic diseases [5]. As miRNAs are present in extracellular fluids such as plasma or serum, urine, saliva, follicular fluid, and semen [30], researchers have analyzed their roles in different cystic pathologies. Thus, the current review aims to discuss the roles of miRNAs in relevant cystic diseases such as polycystic ovarian syndrome (PCOS), polycystic kidney disease (PKD), and pancreatic cyst tumors (PCTs) and their use as potential biomarkers for the prognosis of such diseases.

## 2. Roles of MiRNAs in Cystic Diseases

### 2.1. Polycystic Ovarian Syndrome

**Figure 2:**
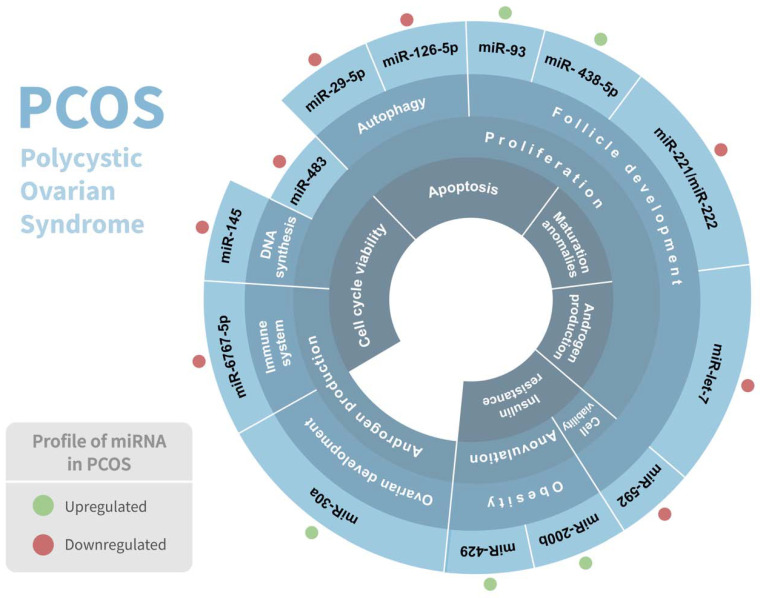
Association of miRNAs in polycystic ovarian syndrome (PCOS) pathophysiology. Regulation of miRNAs in PCOS and their respective biological response involved are shown. Red and green dots indicate the differential expression of each miRNA.

Polycystic ovarian syndrome (PCOS) is the most common endocrine disorder among women of reproductive age, affecting between 5 and 15% of them, and is considered a complex genetic disorder with a multifactorial etiology that involves numerous genes [31]. It is considered a heterogeneous condition since it has numerous phenotypes. PCOS is distinguished by clinical and/or biochemical hyperandrogenism, chronic anovulation, polycystic ovaries, and follicle abnormalities, and is frequently associated with hyperlipidemia, elevated blood insulin levels, type 2 diabetes, inflammation, chronic anovulation, and insulin resistance [32,33]. Although PCOS has been linked to abnormal carbohydrate, lipid, and protein metabolism [34], the genetic etiology and underlying metabolic pathway of PCOS are still not very well elucidated. Intriguingly, several studies have demonstrated a significant association of various miRNAs in PCOS pathophysiology (Table 1) (Figure 2).

In PCOS patients, dysfunctional granulosa cells (GCs) with a low apoptotic and significant proliferation rate have been associated with ovarian dysfunction and abnormal folliculogenesis, which is the principal cause of anovulation [35,36,37]. In this context, Cai et al. [35] noticed a significant downregulation of miR-145 in the isolated human GCs from aspirated follicular fluid of women with PCOS. Interestingly, when upregulated, miR-145 inhibited cell survival rate, DNA synthesis, and proliferation by targeting the insulin receptor substrate 1 (IRS1) and consequently inhibiting the MAPK/ERK signaling pathway. Therefore, this miRNA could be used to improve GCs dysfunction in PCOS, offering a possible solution to anovulation problems presented by PCOS patients [35].

Additionally, in both human GCs and rat ovarian tissue PCOS models, a significant downregulation of miR-126-5p and miR-29a-5p, along with overexpression of its target, the klotho protein, and subsequent suppression of insulin growth factor 1 (IGF-1R) and Wnt family member 1 (Wnt1) were observed [38]. Klotho participates in cell proliferation, apoptosis, and autophagy by inhibiting several signaling pathways, including the IGF-1, fibroblast growth factor (FGF), and Wnt/ß catenin [39]. Furthermore, knocking down the klotho gene in GCs from PCOS patients boosted IGF-1R and Wnt1 protein expression as well as Akt phosphorylation, which was linked to lower insulin-induced apoptosis and higher cell proliferation in human GCs. On the other hand, in the ovarian tissues of PCOS rats, the klotho protein was also found to have a regulatory effect on IGF-1R and Wnt1 protein production as well as Akt phosphorylation. Thus, klotho might play a role in GC apoptosis by modulating the insulin/IGF-1, Wnt1, and Akt signaling pathways. Furthermore, Mao et al. [38] revealed that the overexpression of the klotho gene is crucial in ovarian GC apoptosis in PCOS and that loss of miR-126-5p and miR-29a-5p expression is an etiological factor; thus, the miR-126-5p, miR-29a-5p/klotho/insulin-IGF-1, Wnt, and Akt signaling pathways may have a role in GC apoptosis and subsequently in PCOS development [38].

Several studies have enlightened the importance of miRNAs in cumulus GCs for ovarian follicle development and PCOS pathophysiology. For example, utilizing immortalized human granulosa-like tumor cell line (KGN), Jiang et al. [36] discovered that miR-93 expression was upregulated in PCOS ovaries, and via targeting CDKN1A, it promotes GCs proliferation and G1 to S transition, possibly contributing to folliculogenesis. Although miRNA-93 serves as a tumor promoter, Jiang et al. [36] proposed this miRNA as a GC proliferation promoter. Moreover, high insulin concentration, which is involved in regulating ovarian function, might downregulate CDK1A by inducing the miR-93 expression and stimulating cell proliferation in GC [36].

Besides, Xu et al. [33] investigated the miRNA expression profiles of cumulus GCs from women with and without PCOS, and they noticed a total of 59 differentially expressed miRNAs between the samples, among which miR-221/miR-222 in PCOS cumulus GCs was the most significantly downregulated one. The diminished expression of miR-221/miR-222 was possibly due to a high serum androgen concentration, which might lead to an overexpression of p27/kip1 and consequently to a decreased proliferation of GCs, which is a hallmark of PCOS, resulting in two crucial symptoms presented by PCOS patients: follicle development and maturation abnormalities [33]. Additionally, miR-483-5p was found to be upregulated in PCOS cumulus GCs, and it was identified to regulate both Notch and MAPK pathways by targeting and downregulating Notch 3 and MAPK3, respectively. Notably, Notch signaling is associated with the formation and development of follicles by regulating proliferation and apoptosis in GCs, while MAPK3 has been associated with the proliferation of GCs through the cAMP-PKA pathway. Importantly, both abnormal folliculogenesis, as well as GCs proliferation and apoptosis, are hallmarks of PCOS. Furthermore, miR-483-5p-Notch3/MAPK3 is hypothesized to be mediated by androgen receptors in GCs of women with PCOS [33].

Likewise, Xiang et al. [40] analyzed the miR-483 expression in the lesion and standard ovarian cortex tissue samples from females with PCOS and KGN cell lines. Results revealed that miR-483 was significantly downregulated in ovarian cortex lesions of PCOS patients; however, its artificial overexpression in KGN cells led to the inhibition of cell viability and proliferation as well as suppression of three cell cycle regulators (CCNB1, CCND1, and CDK2) that triggered S or G2/M arrest in transfected cells. In contrast, miR-483 knockdown triggers the viability of KGN cells. Moreover, IGF1, an insulin-linked peptide targeted by miR-483, was investigated as it has been shown that insulin sensitivity is more prone to be decreased by the body fat in PCOS patients, implying a greater demand for insulin to satisfy metabolic needs. Results showed that miR-483 regulates KGN cell viability and proliferation through IGF1 and could be a potent biomarker for PCOS prognosis.

Several studies have been performed analyzing serum miRNAs, as they are highly specific and stable [23]. For the first time, Long et al. [41] analyzed the serum miRNA expression profiles of patients diagnosed with PCOS, and they reported a significant upregulation of miR-222, miR-30c, and miR-146a. Bioinformatic analysis revealed that a number of target genes of the aforesaid miRNAs are actively involved in cell cycle, metastasis, apoptosis, and endocrine pathways targeting mainly P13k-Akt, MAPK, and Toll-like receptor signaling pathways. They also showed that miR-222 has a positive association with serum insulin, while miR-146 is negatively associated with serum testosterone; both insulin and testosterone levels have been altered in PCOS patients. Therefore, these miRNAs could be used as potential biomarkers for the diagnosis of PCOS.

Likewise, Song et al. [42] noticed a significant downregulation of serum miR-592 levels in patients with PCOS. Notably, this miRNA was inversely correlated with the serum levels of luteinizing hormone/chorionic gonadotropin receptor (LHCGR), a significant target of miR-592, which regulates follicle development. In steroidogenic human KGN, the overexpression of miR-592 inhibited cell viability and the G1 to S transition, whereas LHCGR co-transfection reversed the inhibitory role of miR-592. Interestingly, this study also stated that miR-592 potentially targets the IGF-1 receptor (IGF1R) gene, which has been associated with markers of insulin resistance [42].

Similarly, Eisenberg et al. [43] noticed an alteration in the miR-200b and miR-429 expression in serum of anovulatory PCOS women in the early follicular phase. These two miRNAs have been demonstrated to target the mammalian reproduction genes ZEB1 and ZEB2, and their levels diminished after gonadotropin stimulation and ovulation induction [44]. Nonetheless, the expression of miR-200b and miR-429 is assumed to be leptin-dependent, and its upregulation has been associated with PCOS anovulation, obesity, and insulin resistance. 

Moreover, Song et al. [45] proposed the miRNA-6767-5p as a novel biomarker candidate in PCOS diagnosis as its serum level was significantly dropped in PCOS patients, and when adjusted for BMI and age, this miRNA was negatively associated with fasting glucose and positively correlated with the number of menses per year in PCOS women. Intriguingly, most of the genes targeted by miRNA-6767-5p were associated with the immune system and cell cycle modulation. In PCOS, the immune system plays an essential role since this disease has been related to inflammation and alteration of ovarian function by macrophages. Additionally, miR-6767-5p expression was found to be positively correlated with sex hormone-binding globulin (SHBG) and negatively associated with free androgen index (FAI), implying that miR-6767-5p play a major role in PCOS hyperandrogenemia. Furthermore, miR-6767-5p is thought to have a function in the metabolic manifestations of PCOS, as it is closely related to fasting glucose and SHBG, which is a predictor of the risk of type 2 diabetes mellitus.

Other research suggested that some of the miRNAs expressed in ovarian follicles can also be found in the bloodstream and other body fluids such as follicular fluid. In this context, Scalici et al. [46] reported that the expression of let-7 and miR-140 were significantly diminished while miR-30a was significantly upregulated in the follicular fluid samples of women with PCOS and that the combination of these miRNAs was linked to PCOS with high sensitivity and specificity. Let-7 has been shown to regulate the TGF-β signaling pathway by targeting the activin receptor I and Smad2/3 genes [47], contributing to reproductive abnormalities in PCOS such as the perturbation of follicle development and hyperandrogenism [48], whereas miR-140, a tumor suppressor miRNA whose downregulation has been proved to be mediated via ERα signaling [49], has been altered in PCOS. Moreover, miR-30 overexpression has been demonstrated to promote BCL2A1, IER3, and cyclin D2 expression through FOXL-2 repression, affecting ovarian development and androgen production, which are two main altered aspects in PCOS [46].

Furthermore, miR-92a and miR-92b, from the miR-17-92 cluster were significantly downregulated in ovarian theca interna tissues of PCOS women [50]. Both the miRNAs were predicted to target the insulin receptor substrate proteins 2 (IRS-2), which is crucial in the insulin signaling pathway and is congruent with the relative hyperinsulinemia of the analyzed PCOS patients [50]. Additionally, miR-92a was also predicted to target GATA-binding factor 6 (GATA6), a gene related to androgen production, possibly suggesting a relationship between insulin and androgenic signaling pathways in PCOS patients [50].

Recently, Butler et al. [51] investigated the miRNA expression pattern in the follicular fluid from anovulatory women with PCOS undergoing in vitro fertilization and a control group without PCOS. They detected 29 differentially expressed microRNA, where miR-381-3p, miR-199b-5p, miR-93-3p, miR-361-3p, miR-127-3p, miR-382-5p, and miR-425-3p were the most significantly upregulated. Noticeably, miR-199b-5p was correlated with anti-mullerian hormone (AMH), which is known to be elevated in PCOS and considered as a biomarker of this disease, while miR-93-3p was associated with C-reactive protein (CRP), an upregulated marker of inflammation in PCOS. 

In sum, all the above information implies the importance of many miRNAs as potential biomarkers for PCOS and could be used as a prognostic tool in the future. However, microRNA-mediated precise biological mechanisms and signaling pathways in PCOS need to be further investigated to develop sensitive and non-invasive miRNA-based tools to diagnose and manage this disease and its various clinical manifestations.

### 2.2. Polycystic Kidney Disease

Polycystic kidney disease (PKD) is a monogenic disorder characterized by fluid-filled cysts in the renal parenchyma and cysts in other epithelial organs [10]. PKD patients exhibit massive bilateral kidney enlargement due to numerous cysts, leading to end-stage renal failure [52]. According to the type of inheritance, PKD is classified as autosomal dominant PKD (ADPKD) or autosomal recessive PKD (ARPKD). ADPKD is usually caused by mutations in the PKD1, PKD2, or GANAB genes [53,54] which encode polycystin 1 (PC1) and polycystin 2 (PC2), respectively [55,56], and leads to excessive proliferation of the renal tubular epithelium, causing cyst formation [57] and progressive renal interstitial fibrosis (RIF) [58]. While ARPKD is caused by mutations in PKHD1, which encodes fibrocystin, primarily affecting neonates and children [59].

**Figure 3:**
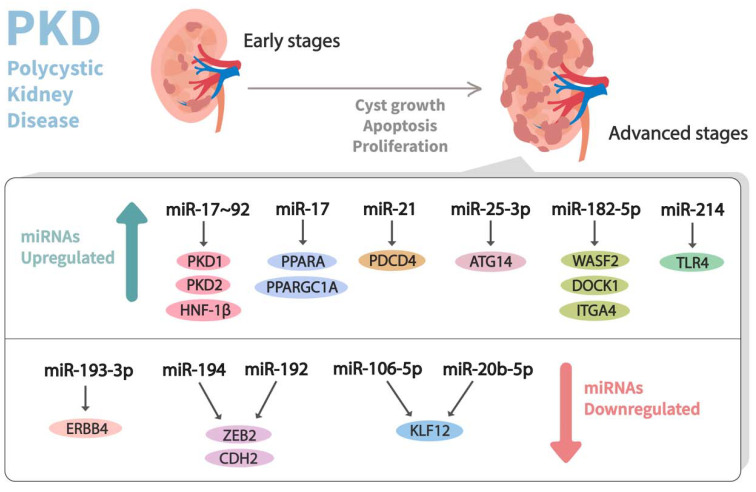
Dysregulation of miRNA expression profiles observed in advanced stages of polycystic kidney disease (PKD). Differential expressions of miRNAs that contribute to the pathogenesis of PKD and their corresponding mRNA targets are shown.

In the past decade, dysregulation of miRNA expression has been observed in PKD (Table 1) (Figure 3). In this milieu, Patel et al. [60] performed a microarray experiment using RNA from the tissues of Kif3a-KO (an animal model of PKD) mice kidneys and healthy control kidneys. They observed that the miR-17-92 miRNA cluster, which primarily encodes miR-17, miR-18a, miR-19a, miR-19b-1, miR-20a, and miR-92a, was significantly upregulated in the aforesaid mouse model of PKD. This cluster was stated to modulate the cystic growth in the kidney by triggering cyst epithelial proliferation and inhibiting the post-transcriptional expression of PKD1, PKD2, and hepatocyte nuclear factor 1β (HNF-1β). Additionally, it might also indirectly affect PKHD1, possibly by the downregulation of its transcriptional activator, HNF-1β. Likewise, to understand the pathogenic role of the miR-17–92 cluster in ADPKD, Hajarnis et al. [61] genetically deleted it from various orthologous ADPKD mouse models, leading to the conclusion that the deletion of this cluster inhibits cystic proliferation and disease progression in early-onset ADPKD models. Furthermore, they demonstrated that miR-17–92 deletions attenuate disease progression in long-lived and slow cystic growth models of ADPKD by improving the expression of mitochondrial and metabolism-related gene networks. Moreover, miR-17 was noticed to be significantly upregulated in kidney cysts of mouse and human ADPKD, and it modulates the mitochondrial function and aggravates cystic growth through direct repression of the peroxisome proliferator-activated receptor α (PPARA).

Nevertheless, Yheskel et al. [62] recently revealed that anti-miR-17 could improve renal function and reduce kidney injury and proliferation of cysts by upregulating mitochondrial metabolism pathways and downregulating the inflammation and fibrosis pathways. Furthermore, within the miR-17 family, Shin et al. [63] found downregulation of miR-20b-5p and miR-106-5p and upregulation of their respective target KLF12 in PKD2 knockout mice kidney tissue. They also determined that KLF12 is involved in regulating cystogenesis since its knockdown retarded cystic growth and cell proliferation. Altogether, their results showed that the miR-17 family constitutes a pathogenic element and the main contributor to the progression of cysts, so its study could provide a new prognostic and therapeutic tool for ADPKD patients [61].

More recently, a plethora of evidence suggests that immune cells in the cystic microenvironment affect ADPKD progression, and as miRNAs play a crucial role in regulating inflammatory and immune responses, there has been growing interest in finding a direct correlation. In this context, Lakhia et al. [64] noticed that miR-214, derived from the long non-coding RNA (lncRNA) DNM3OS, is upregulated in mouse and human PKD. Their results also suggested that the cyst microenvironment is affected by the genetic deletion of miR-214, leading to increased inflammation and cystic growth in a slowly progressive ADPKD model. Considering miR-214 removal augments macrophage accumulation in the cystic environment, they speculated that miR-214 might function to restrain inflammation signaling pathways. It was also found that miR-214 directly inhibits TLR4, which raises the possibility of miR-214 functioning as a negative feedback loop to restrain the TLR4 signaling pathway and limit the maladaptive response and disease progression. 

Apoptosis and autophagy are essential molecular processes that maintain organic and cellular homeostasis. In recent years, it has been observed that numerous miRNAs have participated in the regulation of both autophagy and apoptosis, and their dysregulation has a direct impact on the pathogenesis of renal cystic growth [65]. For example, Lakhia et al. [66] elucidated a novel mechanism involving miRNAs and the regulation of proapoptotic genes in the pathogenesis of cystic kidney growth. They found that miR-21 transcript was upregulated in murine models of PKD and human ADPKD, and its expression was associated with cystic expansion, particularly in tubular cysts in kidney samples. Their results also demonstrated that miR-21 promoted cystic growth, possibly through direct repression of Programmed cell death 4 (PDCD4), a novel tumor suppressor that promotes apoptosis. Moreover, they noticed that cAMP signaling was a direct cause for the transactivation of miR-21 in kidney epithelial cells as it activated protein kinase A (PKA), which phosphorylated cAMP response element-binding protein (CREB) and promoted its transcriptional activity. Furthermore, they stated that by deleting miR-21, cystic growth could be attenuated in mouse models of ADPKD. In conclusion, their results suggested that the anti-miR-21 strategy might serve as a new therapeutic approach for PKD. 

Liu et al. [67] revealed a significant upregulation of miR-25-3p in mice models of PKD. It was later demonstrated through in vivo and in vitro experiments that inhibition of miR-25-3p increased autophagy in renal cells, thus repressing cystic formation in PKD. Similarly, Streets et al. [68] discovered that the downregulation of miR-193-3p triggers the expression of ERBB4 in human ADPKD and in PKD1 mouse models, which suggests that ERBB4 might be a target of miR-193-3p. They also found that ERBB4 was principally expressed as two different isoforms (JMa-CYT-1 and JMa-CYT-2) in kidney tissue and ADPKD cystic cells. The augmentation of its expression was associated with increased ligand-induced activated signaling; as a result, cystic expansion and proliferation were observed, thus suggesting its involvement in the pathology of cystogenesis. 

The extracellular matrix (ECM) is essential for tissue growth and homeostasis since it provides structural and mechanical integrity to the surrounding cells while also helping to efficiently regulate the cell microenvironment. Interestingly, miRNAs have an important role in the development, maintenance, and modification of the ECM [69]. As ECM remodeling and basement membrane abnormalities are common hallmarks of cysts in human ADPKD and ARPKD [70], investigations to understand their correlation have been carried out. In this regard, Kim et al. [2] discovered that miR-192 and miR-194 expression was downregulated in ADPKD mouse models and human patients through the process of hypermethylation at their respective loci. They also noticed that miR-192 and miR-194 directly target ZEB2 and CDH2 genes, which play a role in cell adhesion and are associated with EMT processes that cause cystic expansion. Moreover, by restoring the expression levels of miR-192 and miR-194 in ADPKD mouse models, cystic growth was reduced, and renal functions were rescued. 

Besides, Woo et al. [71] revealed that upregulated miR-182-5p is involved in actin cytoskeleton signaling and cystic progression in PKD1 conditional knockout mice. Their results also suggested that although the inactivation of PKD1 and PKD2 have similar processes during cystic formation, their cystic expansion procedures are different. This concurs with their findings that miR-182-5p suppressed WASF2, DOCK1, and ITGA4 only in PKD1-deficient mice, causing defects in the actin cytoskeleton organization, thus promoting cyst enlargement. Moreover, among the direct targets of miR-182-5p, WASF2 is essential for the actin regulatory machinery and helps mediate the actin cytoskeletal organization; thus, its repression promotes cystic growth. Additionally, DOCK1 is an exchange factor that contributes to the formation of lamellipodial protrusions, and its suppression was shown to stimulate cystic development. 

All the evidence presented above suggests that the alterations in the expression of specific miRNAs contribute to the pathogenesis of PKD, signifying that these small molecules could be used as biomarkers for disease progression as well as novel therapeutic targets.

### 2.3. Pancreatic Cyst Tumors

**Figure 4:**
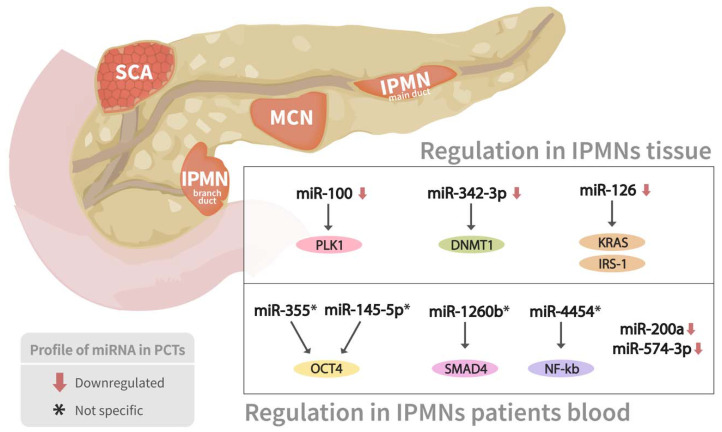
miRNAs expressions profile in two types of pancreatic cyst tumors. Association between several miRNAs’ expression profiling in both branch duct papillary mucinous neoplasms (BD-IPMN) and main duct IPMN, as well as their corresponding mRNAs targets, are shown.

Pancreatic cyst tumors (PCTs) are a heterogeneous group of tumors that account for 2 to 10% of pancreatic lesions and range in severity from benign to malignant [72]. The three most frequent kinds of PCTs are intraductal papillary mucinous neoplasms (IPMNs), mucinous cystic neoplasms (MCNs), and serous cystic neoplasms (SCNs), which account for almost 90% of all PCTs [72]. miRNA expression profiling has shown considerable promise in identifying pathologic characteristics, malignant transformation, biologic activity, and molecular evolution of PCTs (Table 1) (Figure 4).

Lee et al. [73] examined a broad array of differentially expressed miRNAs for designing an accurate disease classifier panel to distinguish between distinct pancreatic cysts. Notably, miRNAs expression profiles were able to identify non-mucinous cysts (serous cystadenoma) from mucinous cysts, branch duct IPMN (BD-IPMN), main duct IPMN (MD-IPMN) neoplasms, and pancreatic ductal adenocarcinoma/pancreatic cancer (PDAC) with diagnostic accuracies reaching >95%. Specifically, a serous cystadenoma (SCA) classifier comprised miR-31-5p, miR-483-5p, miR-99a-5p, and miR-375 could distinguish SCA from all other mucinous cystic lesions, including MCN, BD-IPMN, MD-IPMN, and PDAC. Similarly, an MCN classifier including miR-10b-5p, miR-202-3p, miR-210, and miR-375 was designed to distinguish MCN from SCA, IPMN, and PDAC accurately. Further, the mucinous group’s distinct miRNA expression pattern was examined. A four-miRNA panel, consisting of miR-192-5p, miR-202-3p, miR-337-5p, and miR-130-3p, was used to differentiate MCN from BD-IPMN with high sensitivity and accuracy. PDAC was also discriminated from IPMN using a PDAC classifier (miR-21-5p, miR-485-3p, miR-708-5p, and miR-375). Undeniably, the miRNA biomarker classifiers can fulfill the evident need for an improved diagnostic tool to manage cystic pancreatic lesions by distinguishing malignant, premalignant, and benign forms, as well as determining which cystic neoplasms are likely to progress to malignancy. 

Permuth-Wey et al. [74] conducted an IPMN tissue-based genome-wide miRNA expression profiling to identify a panel of miRNAs that accurately classify IPMN risk status from 28 surgically excised, pathologically proven IPMNs. Six miRNAs (miR-100, miR-99b, miR-99a, miR-342-3p, miR-126, and miR-130a) were shown to be considerably downregulated in high-risk IPMNs compared to low-risk IPMNs, implying that low expression levels of these miRNAs might be linked to PDAC invasion progress.

Intriguingly, tumor suppressors and regulators of PDAC development are among the targets of dysregulated miRNAs. For example, Polo-like kinase 1 (PLK1), a critical target of miR-100, can prevent cancer progression and regulate the proliferative activity in early PDAC when overexpressed. Furthermore, the DNA methyltransferase 1 (DNMT1) gene was discovered to be a validated target gene of miR-342-3p, which maintains DNA methylation and has been linked to PDAC progression. Besides, miR-126 has been linked to the development of PDAC by targeting oncogenes such as KRAS and insulin receptor substrate-1 (IRS-1), a mediator of phosphoinositide 3-kinase (PI3K) activation in quiescent PDAC cells. This research highlighted new miRNAs that may help predict the severity of IPMNs and shed light on miRNA-mediated pancreatic malignancy progression. In another similar study, Permuth-Wey et al. [75] analyzed plasma miRNA expression levels specifically in individuals newly diagnosed with IPMNs and healthy controls and discovered a 30-miRNA gene pattern with 2- to 4-fold higher expression in IPMN cases compared to controls. Markedly, miR-145-5p and miR-335, which target the transcription factor OCT4, may be implicated in suppressing cancer stem cell features of PDACs.

Moreover, a significantly lower expression of a five-miRNA signature (miR-200a-3p, miR-1185-5p, miR-33a-5p, miR-574-3p, and miR-663b) was also noticed in malignant IPMNs compared to benign cases. Remarkably, the downregulation of miR-200a has been linked to epithelial-to-mesenchymal transformation and early metastasis. In contrast, decreased expression of miR-574-3p, which functions as a tumor suppressor and regulates critical cell signaling pathways like Jak-STAT and Wnt/-catenin involved in carcinogenesis, could regulate several oncogenes that promote malignant IPMN status. The biological plausibility of their findings suggested that the discovered miRNAs could impact critical pathways implicated in PDAC development and progression [75].

Altogether, a significant association of several miRNAs has been noticed in various types of PCTs, which could be used as potential novel biomarkers and diagnostic tools.

**Table 1:** miRNAs involved in cystic diseases.

Disease	miRNA	Target	Biological Mechanism	Source	Reference
PCOS	miR-145 ↓	IRS1	Cell survival, DNA synthesis, proliferation	Human GCs from aspirated follicular fluid	[35]
	miR-126-5p ↓	Klotho gene	Apoptosis, proliferation, autophagy	Human GCs and rat ovarian tissue	[38,39]
	miR-29a-5p ↓				
	miR-93 ↑	CDKN1A	Proliferation, G1 to S transition, folliculogenesis	Human GCs	[36]
	miR-221/miR-222 ↓	p27/kip1	Proliferation, follicle development, maturation abnormalities	Cumulus GCs	[33]
	miR-438-5p ↑	Notch 3, MAPK3	Follicle formation and development, proliferation, apoptosis		
	miR-483 ↓	IGF1	Cell viability, proliferation, cell cycle arrest	Ovarian cortex tissue and KGN	[40]
	miR-222 ↑	P13k-Akt, MAPK, Toll-like receptors	Cell cycle, metastasis, apoptosis, endocrine pathways	Serum	[41]
	miR-30c ↑				
	miR-146a ↑				
	miR-592 ↓	LHCGR, IGF-1	Follicle development, cell viability, cell cycle progress, insulin resistance	Serum	[42]
	miR-200b ↑	ZEB1, ZEB2	Reproduction, anovulation, obesity, insulin resistance	Serum of anovulatory women	[43,44]
	miR-429 ↑				
	miRNA-6767-5p ↓	No reports	Immune system, cell cycle, hyperandrogenemia	Serum	[45]
	let-7 ↓	Activin receptor I, Smad2/3	Follicle development, hyperandrogenism	Follicular fluid	[46,47,48,49]
	miR-140 ↓	No reports			
	miR-30a ↑	FOXL-2	Ovarian development, androgen production		
	miR-381-3p, miR-199b-5p, miR-93-3p, miR-361-3p, miR-127-3p, miR-382-5p, miR-425-3p *	No reports		Follicular fluid from anovulatory women undergoing in vitro fertilization	[51]
PKD	miR-17~92 cluster ↑	PKD1, PKD2, HNF-1β	Proliferation, disease progression	Mice kidney tissue	[60,61]
	miR-17 ↑	PPARA PPARGC1A	Cyst proliferation, PKD progression	Mice PKD1-KO kidneys, PKD2-KO	[61,62]
	miR-20b-5p ↓	KLF12	Cystogenesis	Mice kidney tissue, embryonic fibroblasts, and cell lines; human ADPKD cell lines	[63]
	miR-106-5p ↓				
	miR-214 ↑	TLR4	Cyst microenvironment	Mice PKD1-KO and PKD2-KO kidneys	[64]
	miR-21 ↑	PDCD4	Apoptosis	Mice PKD1-KO and PKD2-KO kidneys	[66]
	miR-25-3p ↑	ATG14	Renal and smooth muscle, proliferation	PKD mice kidney tissue	[67]
	miR-193-3p ↓	ERBB4	Ligand induced-activated signaling	Epithelial cells from ADPKD human kidneys	[68]
	miR-192 ↓ miR-194 ↓	ZEB2 CDH2	Cell adhesion, EMT processes	Renal cyst tissue from ADPKD patients	[2]
	miR-182-5p ↑	WASF2	Actin cytoskeletal organization	Kidney samples from PKD1- and PKD2-deficient mice	[71]
		DOCK1	Lamellipodial protrusions		
		ITGA4	Migratory events		
PCTs	miR-100 ↓	PLK1	Cancer progression, proliferation	IPMNs tissue	[74]
	miR-342-3p ↓	DNMT1	PDAC progression		
	miR-126 ↓	KRAS IRS-1	PI3K activation		
	miR-145-5p *	OCT4	Cancer cell stems properties	Blood from IPMNs patients	[75]
	miR-355 *				
	miR-1260b *	SMAD4	Key drivers in pancreatic carcinogenesis		
	miR-4454 *	NF-kb			
	miR-200a ↓	No reports	Epithelial-to-mesenchymal transformation, early metastasis		
	miR-574-3p ↓	No reports	Malignant IPMN status		

↑ Indicates upregulated; ↓ Indicates downregulated; * Dysregulation not specified.

## 3. Conclusions

In recent years, the study of miRNAomics has illuminated our understanding of many complex diseases due to their stability and easy identification in body fluids and tissues. This characteristic facilitates the detection and characterization of disease-specific miRNAs, their targets, and their relation to pathophysiology.

These molecules can be used to detect the presence of any disease pathology as well as its stages, progression, and genetic origin. However, as a single miRNA might have hundreds of mRNA targets and several miRNAs can control a single target, the complex interplay between particular miRNAs and functionally targeted genes must be thoroughly examined. In some cases, a single miRNA biomarker may be enough to predict a health outcome; however, in others, a well-defined panel of miRNAs is required for better diagnostic sensitivity and/or specificity. Moreover, caution is required when the selected biomarker is known to be ubiquitously expressed. For example, some miRNAs have been frequently increased or decreased in individuals with diverse conditions. Nonetheless, a rising number of studies have indicated that subsets of miRNAs can be utilized as biomarkers in various disorders [76], and the list of miRNAs that could be employed as biomarkers is extensive [77]. In this sense, many clinical trials have been completed or are currently being conducted to test new miRNA-based diagnostic and prognostic panels [78].

Furthermore, miRNAs are also attractive pharmacological agents for modulating the abundance of target mRNAs/proteins and pathways of interest due to their small size and low antigenicity. Synthetic sequence-specific oligonucleotides that mimic or suppress miRNA expression are used in most miRNA-based therapeutic studies. As FDA-approved the use of small RNA drugs in clinical medicine, ongoing studies for the miRNA-based prospective medications are either in preclinical studies or in phase 1 and phase 2 clinical trials. For example, RGLS4326 (the only miRNA-based drug currently available in clinical settings to treat cystic disease) is a first-in-class anti-miR-17 oligonucleotide with promising potential for treating PKD evidencing favorable potency, stability, safety, pharmacokinetics, and pharmacodynamic characteristics [57]. Furthermore, Miravirsen and RG-101 (miRNA inhibition therapy for hepatitis C) [79,80], MRX34 (miRNA mimic for cancer therapy) [81] are some of the most promising therapies based on miRNA technologies. As a result, investors are becoming more interested in this field of research, and numerous pharmaceutical companies have started to develop miRNA-based therapeutics (e.g., miRagen Therapeutics Inc., Mirna Therapeutics Inc., and Santaris Pharma) [82,83]. However, various obstacles must be overcome, including safe and organ-specific delivery and long-term efficacy. Pending the resolution of these concerns, it is reasonable to expect that miRNA-targeting drugs will eventually be introduced into clinics. 

Although the dysregulated gene expression of every cystic disease is different, miRNAs have been associated with a vast number of molecular processes such as abnormal cell proliferation, apoptosis, and metabolic perturbation that contribute to cystic formation. Therefore, miRNAs have emerged as potential prognostics tools and biomarkers for cystic diseases, providing a molecular perspective of this complex group of diseases. However, despite the tremendous and significant advances made in this area, there is still a long way to enlighten the detailed miRNA-mediated pathophysiology of cystic diseases to develop more accurate functional biomarkers and highly reliable miRNA-based therapeutic drugs.

## Data Availability

Not applicable.

## Abbreviations

miRNA	microRNA
pri-mRNA	Primary miRNA
DGCR8	DiGeorge syndrome critical region 8
pre-miRNA	Precursor miRNA
RISC	RNA-Induced Silencing complex
AGO	Argonaute
PCOS	Polycystic ovarian syndrome
PKD	Polycystic kidney disease
PCTs	Pancreatic cyst tumors
GCs	Granulosa cells
IRS1	Insulin receptor substrate 1
MAPK	Mitogen-activated protein kinase
IGF-1R	Insulin growth factor 1
Wnt1	Wnt family member 1
DHEA	Dehydroepiandrosterone
CDKN1A	Cyclin-dependent kinase inhibitor 1A
cAMP	Cyclic adenosine 3′, 5′-monophosphate
PKA	Protein kinase A
CCNB1	Cyclin B1
CCND1	Cyclin D1
CDK2	Cyclin-dependent kinase 2
LHCGR	Luteinizing hormone/chorionic gonadotropin receptor
IGF1R	IGF-1 receptor
ZEB1	Zinc Finger E-Box Binding Homeobox 1
ZEB2	Zinc Finger E-Box Binding Homeobox 2
BMI	Body mass index
SHBG	Sex hormone-binding globulin
FAI	Free androgen index
IRS-2	Insulin receptor substrate proteins 2 GATA6 GATA-binding factor 6
CCs	Cumulus cells
RUNX2	Runt-related transcription factor 2
TGF-β	Transforming growth factor-β
ERα	Estrogen receptor α
IER3	Immediate Early Response 3
AMH	Anti-mullerian hormone
CRP	C-reactive protein
ADPKD	Autosomal dominant PKD
ARPKD	Autosomal recessive PKD
GANAB	Glucosidase II α subunit
PC1	Polycystin 1
PC2	Polycystin 2
RIF	Renal interstitial fibrosis
HNF-1β	Hepatocyte nuclear factor 1β
PPARA	Peroxisome proliferator-activated receptor α
KGN	human granulosa-like tumor cell line
KLF12	Kruppel like factor 12
lncRNA	Long non-coding RNA
TLR4	Toll-like receptor 4
PDCD4	Programmed cell death 4
CREB	cAMP response element-binding protein
ATG14	Autophagy related 14
PCNA	Proliferating Cell Nuclear Antigen
MYH7B	Myosin Heavy Chain 7B
CDH2	Cadherin 2
EMT	Endothelial–mesenchymal transition
DOCK1	Dedicator of cytokinesis 1
ITGA4	Integrin subunit α 4
ERBB4	Erb-B2 receptor tyrosine kinase 4
IPMNs	Intraductal papillary mucinous neoplasms
MCNs	Mucinous cystic neoplasms
SCNs	Serious cystic neoplasms
PDAC	Pancreatic ductal carcinoma
PCF	Pancreatic cystic fluid
PLK1	Polo-like kinase 1
DNMT1	DNA methyltransferase 1
PI3K	Phosphoinositide 3-kinase
OCT4	Octamer-binding transcription factor 4
SIP1	Smad interacting protein
